# Evaluation of bone thickness at infra-zygomatic crest region compared with cervical vertebrae maturation index

**DOI:** 10.6026/973206300200630

**Published:** 2024-06-30

**Authors:** Shabdika Baghel, Anila Rupa Kujur, Binita Venu Gopal, Jyoti Kumari, Alok Kumar Gupta, Vinit Kumar Singh

**Affiliations:** 1 Department of Orthodontics & Dentofacial Orthopedics, Sri Aurobindo College of Dentistry, Indore, M.P., India; 2 Department of Orthodontics & Dentofacial Orthopedics, Vananchal Dental College, Farathiya Garhwa, Jharkhand, India

**Keywords:** Infrazygomatic arch, bone thickness, CVMI stages

## Abstract

Orthodontists should know variation in thickness of infrazygomatic crest region according to maturation status of patients. Therefore, it is of interest to evaluate the thickness of bone at infrazygomatic crest region and to correlate the thickness of bone with cervical vertebrae maturation index (CVMI) incorporating CBCT.A retrospective analysis of 120 patients' CBCT scans60 of them male and 60 female-was carried out. The thickness of the bone was determined at five locations. Using CBCT, the cervical vertebral maturation was created and the Hassel-Farmann index was used for analysis. A lone researcher conducted all of the measurements. Bone thickness of infrazygomatic arch at all five locations was found to increase as the maturation stage progressed from initiation stage to maturation stage. Then there was decrease in the bone thickness in completion stages compared to maturation stage. The thickness of bone at infrazygomatic arch is significantly correlated with CVM stages as determined by CBCT.

## Background:

Since the beginning of twentieth century, anchorage has been a crucial factor in orthodontic treatment [1, 2-3]. Conventional anchorage reinforces anchorage with intraoral as well as extraoral techniques such as headgear along with intermaxillary elastics [4, 5-6]. Since cortical anchoring offers more anchorage management with the least amount of patient cooperation, it has supplanted traditional methods in modern times [5, 7-8]. Miniplates, mini-implants and miniscrews are examples of temporary anchorage devices that are widely utilized due to their tiny size, affordability, and simplicity of usage [9, 10-11]. The primary stability of the miniscrews has been evaluated by a variety of parameters, including thickness of bone, design of implant, patient age and torque, appropriate mechanical characteristics of the screws, material employed, and the form and duration of dynamic loading [10-12]. Extensive research is being done to determine safe zones where miniscrews can be inserted without running the danger of damaging tooth roots or irritating mucous tissues [13, 14, 15, 16-17]. Miniscrew risk factors are reduced by employing a variety of techniques, including the use of insertion guides and the measurement of bone thickness using computed tomography (CT) and cone beam computerized tomography (CBCT) [12-14]. According to a study, greater than one mm of cortical thickness is necessary for the implants to be stable. However, the use of interradicular implants for serious malocclusions has declined due to greater likelihood of failure from peri-implantitis, poorer stability under load, and a higher likelihood of root injury [11-18]. To get over these drawbacks, extra alveolar locations such the buccal shelf area and infrazygomatic crest can be utilized. A bony ridge called the infrazygomatic crest lies between the maxilla's alveolar process and zygomatic process. Its bicortical plates allow for accurate regulation of anchorage for efficient orthodontic tooth movement as well as other orthodontic treatments [10- 16].Mini-implants positioned in the infrazygomatic crest do not impede the alignment of orthodontic teeth because they are positioned higher from the root area [9-15]. Nevertheless, because of their proximity to the maxillary antrum and, in younger patients, the mesiobuccal root of the first molar of maxilla, precise measurement of bone thickness is required in order to select the best implants [16- 19]. Numerous studies have been carried out to assess the thickness of intraradicular bone but very few to assess the thickness of infrazygomatic bone. A study indicated that average infrazygomatic thickness of bones is only 1.44 to 1.58 mm [20, 21, 22, 23-24].Many investigations were done using CT, although the main drawback of CT is its expensive nature and increased exposure to radiation [21-25]. Therefore, it is of interest to evaluate the thickness of bone at infrazygomatic crest region and to correlate the thickness of bone with cervical vertebrae maturation index (CVMI) incorporating CBCT.

## Methods and Materials:

A retrospective analysis of 120 patients' CBCT scans-60 of them male and 60 female-was carried out. Each patient radiograph was assigned a unique identity code, and the patients' identities remained a secret. Kodak 9500 CBCT equipment was utilized in this investigation. The configurations were as follows: The parameters that were used were isotropic voxel size of 0.2 mm, spatial resolution of 10 line pairs per centimetre, field of view of 18 x 21 cm, 10 mA, exposure length of 15 s and 90 kVp, voltage. The patients' ages varied between 8 years to 25 years old (Table 1).

## Qualifications for inclusion:

[1] A permanent first molar without a bone lesion

## Criteria for exclusion:

[1] The existence of any tumors,

[2] An atrophic bone present

[3] Cleft lip and palate present

[4] Diseases connected to bone metabolism are present

[5] Teeth that are impacted in the infrazygomatic area

[6] Patients with some missing teeth.

There were two planes on the infrazygomatic crest: the horizontal plane and the vertical plane. The vertical plane travelled through the most anterior region of the infratemporal fossa corresponding to the midsagittal plane, and the horizontal plane ran through the most inferior boundary of the maxillary zygomatic process. In both the horizontal planes and vertical planes, five parallel lines have been established at intervals of two millimeters. At the junction of these lines, the thickness of the bone was determined at five locations (L1,L2,L3,L4 and L5).Using CBCT, the cervical vertebral maturation was created and the Hassel-Farmann index was used for analysis. A lone researcher conducted all of the measurements.

## Statistical analysis:

The relationship between the cervical vertebrae development phases and the overall thickness of the infrazygomatic bone was examined using the Kruskal-Wallis analysis of variance (ANOVA) test. Thickness of bone was expressed in the form of means of bone thickness at different locations and standard deviations. SPSS version 21 was used for statistical analysis. P value ≤0.01 was considered statistically significant.

## Results:

It was observed that bone thickness of infrazygomatic arch at all five locations was found to increase as the maturation stage progressed from stage 1 to stage 5. Then there was decrease in the bone thickness in stage 6 as compared to stage 5. The bone thickness was maximum at L1 (super lateral surface of infrazygomatic arch) corresponding to zygomatic process of maxilla while the minimum thickness was observed at L5 (anterior wall of maxillary antrum) (Table 2). It was observed that bone thickness of infrazygomatic arch was statistically correlated to CVM stage of maturation.

## Discussion:

Since they are positioned higher from the root area, mini-implants placed in the infrazygomatic crest do not obstruct the alignment of orthodontic teeth [15-19]. However, accurate evaluation of bone thickness is necessary to choose the optimal implants due to their close proximity to the maxillary antrum and, in younger patients, the mesiobuccal root of the first molar of the maxilla [20-24]. There are a large number of studies that evaluate the thickness of intraradicular bone, but relatively few that evaluate the thickness of infrazygomatic bone. According to a study, bones' typical infrazygomatic thickness ranges from 1.44 to 1.58 mm [14-21].CT has been used for many examinations, but its primary disadvantages are greater radiation exposure and cost [14-20]. Orthodontists should be aware of how a patient's maturation status affects the diversity in thickness of the infrazygomatic crest region [21-25].This study was therefore conducted to evaluate the thickness of bone at infrazygomatic crest region and to correlate the thickness of bone with cervical vertebrae maturation index (CVMI) incorporating CBCT. This study found that bone thickness of infrazygomatic arch at all five locations was found to increase as the maturation stage progressed from stage 1 to stage 5. Then there was decrease in the bone thickness as compared to stage 5. The bone thickness was maximum at L1 (superolateral surface of infrazygomatic arch) corresponding to zygomatic process of maxilla while the minimum thickness was observed at L5 (anterior wall of maxillary antrum). It was observed that bone thickness of infrazygomatic arch was statistically correlated to CVM stage of maturation.

This can be linked to the maxillary sinus's evolution into a reverse pyramidal shape, which causes enlargement laterally at the upper region, as well as the rise in bone density that occurs with aging [13-18]. The thickness of the infrazygomatic bone varied significantly between the beginning and completion stages, ranging from roughly 0.5 mm to 10 mm [14-21].The findings of our study have similarity with findings of other studies [18-25]. A study indicates that a 1- to 2-mm thickness of infrazygomatic bone is sufficient for the insertion of 4- to 5-mm miniscrews to hold a 2-mm miniplate [12-16]. On the other hand, using a 5- to 7mm-long miniscrew during the early phases of bone growth could cause the maxillary sinus's Schneiderian membrane to puncture. Thus, choosing the best miniscrews for orthodontic purposes requires a precise measurement of bone thickness [17- 24]. An important consideration in orthodontic treatment has been anchoring since the early 2000. Conventional anchorage uses intermaxillary elastics and headgear, among other intraoral and extraoral procedures, to reinforce anchorage [11-20]. In the present era, cortical anchoring has replaced older approaches since it provides more anchorage management with the least amount of patient involvement [10- 16]. Due to their small size, low cost, and ease of use, miniplates, mini-implants, and miniscrews are a few types of temporary anchorage devices that are frequently used [22-25]. Numerous factors have been considered in assessing the primary stability of the miniscrews: bone thickness, implant design, patient age, torque, material used, screw suitability, and the kind and duration of dynamic loading [16- 23]. A great deal of study is being done to identify safe zones where miniscrews can be put without having to worry about hurting mucosal tissues or destroying tooth roots. Numerous strategies are used to lower the risk factors associated with miniscrews, such as using insertion guides and measuring bone thickness with CBCT and CT scans [18- 25].

In our investigation, the thickness of bone also rose in a caudocranial direction, which was consistent with the findings of study which found that the zygomatic bone was 9.8 mm near its edge, while the apical portion had the thinnest bone, measuring 2.7 mm [14-22]. The benefit of miniscrews positioned at the zygomatic process position is less movable mucosa and less hindrance with the movement of the tooth. That study concluded that 5-mm miniscrews should be positioned adjacent to the alveolar process; whereas 7-mm miniscrews should be positioned closer to the zygomatic process [13-21]. A study found that for the implants to be stable, the cortical thickness must be larger than one millimeter [19-22]. However, because of a higher risk of root injury, poorer stability under load, and peri-implantitis failure, the use of interradicular implants for serious malocclusions has decreased. Extra alveolar sites like the buccal shelf area and infrazygomatic crest can be used to overcome these disadvantages [23-25]. Between the zygomatic process and alveolar process of the maxilla is a bony ridge known as the infrazygomatic crest. In addition to traditional orthodontic procedures, its bicortical plates enable precise anchoring adjustment for effective orthodontic tooth movement [12-16].

## Conclusion:

The thickness of bone at infra-zygomatic archs significantly correlated with CVM stages as determined by CBCT.

## Figures and Tables

**Table 1:** Distribution of study participants in each CVM stage with chronological age

**CVM stages**	**Initiation (stage 1)**	**Acceleration (stage 2)**	**Transition (stage 3)**	**Deceleration (stage 4)**	**Maturation (stage 5)**	**Completion (Stage 6)**
**n**	**20**	**20**	**20**	**20**	**20**	**20**
Chronological Age (Years)	8-10	10-12	12-14	13-15	14-17	16-25
Male (n)	10	10	10	10	10	10
Female (n)	10	10	10	10	10	10
In each CVM stage, 20 study participants were there consisting of 10 males and 10 females (Table 1)

**Table 2:** Mean thickness of bone at different locations of infrazygomatic arch in all CVM stages of maturation

	**L1**	**L2**	**L3**	**L4**	**L5**
Initiation (Stage 1)	2.8 ±0.050	1.9± 0.987	1.4 ± 0.934	0.8 ± 0.594	0.6 ± 0.638
Acceleration (Stage 2)	5.3 ± 1.144	3.9 ± 1.330	2.9 ± 0.020	2.0 ± 0.679	1.2 ± 0.427
Transition (Stage 3)	7.4 ± 1.27	6.4 ± 1.363	3.9 ± 1.144	3.1 ± 0.927	2.7 ± 0.954
Deceleration (Stage 4)	7.9 ± 0.890	7.0 ± 0.005	5.2 ± 0.679	4.0 ± 0.679	3.4 ± 0.594
Maturation (Stage 5)	16.5 ± 0.954	8.9 ± 0.743	8.4 ± 0.786	6.6 ± 0.638	6.0 ± 0.679
Completion (Stage 6)	9.0 ± 0.849	8.6 ± 0.083	6.5 ± 0.077	6.1 ±0.927	6.1 ± 0.054
P value	0.001	0.001	0.001	0.001	0.001
